# Feasibility of *In-Vivo* Simulation of Acute Hemodynamics in Human Atrial Fibrillation

**DOI:** 10.1371/journal.pone.0165241

**Published:** 2016-10-20

**Authors:** Marek Sramko, Dan Wichterle, Josef Kautzner

**Affiliations:** Department of Cardiology, Institute for Clinical and Experimental Medicine (IKEM), Prague, Czech Republic; University of Minnesota, UNITED STATES

## Abstract

This study evaluated hemodynamic feasibility and reproducibility of a new method for in vivo simulation of human atrial fibrillation (AF). The method was tested during sinus rhythm in 10 patients undergoing catheter ablation for AF. A simple electronic device was assembled that allowed triggering a cardiac stimulator by predefined series of RR intervals. Irregular RR interval sequences with a mean heart rate of 90/min and 130/min were obtained from ECG recordings of another patients with AF. Simultaneous atrioventricular pacing was delivered by catheters placed inside the coronary sinus and at the His bundle region. Hemodynamic effect of the simulated AF was assessed by invasive measurement of the left ventricular (LV) pressure, dP/dt, and Tau. Compared to regular pacing at the same mean heart rate, the simulated AF significantly impaired the LV both systolic and diastolic function. Repeated AF pacing in the same patients generated similar LV hemodynamics. The proposed method provides a realistic and reproducible in-vivo model of AF. It can be exploited for investigation of the hemodynamic consequences of AF in various patient populations.

## Introduction

Atrial fibrillation (AF) is a common clinical arrhythmia. It can have various hemodynamic consequences. Excessive heart rate, loss of left atrial kick, and heart rhythm irregularity can all impair the cardiac performance [1–3]. Moreover, the ensuing beat-to-beat variations in the systemic blood pressure can dysregulate cardiovascular autonomic modulations and autoregulation of blood flow in central organs [4–6]. However, little is known about the individual role of each of these hemodynamic consequences of AF and their clinical implications in different patient populations. One of the reasons is the absence of an appropriate hemodynamic model of AF.

We developed a new pacing-based method for simulation of the hemodynamics in AF in vivo. Compared to previously used methods [2–4, 7, 8], our method is applicable to any patient population, uses more physiological His pacing, allows to reproduce virtually any RR interval pattern recorded from a patient with AF, and allows to individually scrutinize diverse hemodynamic aspects of AF.

In this study we evaluated the feasibility of the method by assessment of the left ventricular (LV) performance during the simulated AF and during regular pacing with matched heart rate. We also evaluated reproducibility of the method by repeated assessment of the LV performance in the same patients.

## Materials and Methods

### Principle of the AF simulation

**Fig 1:**
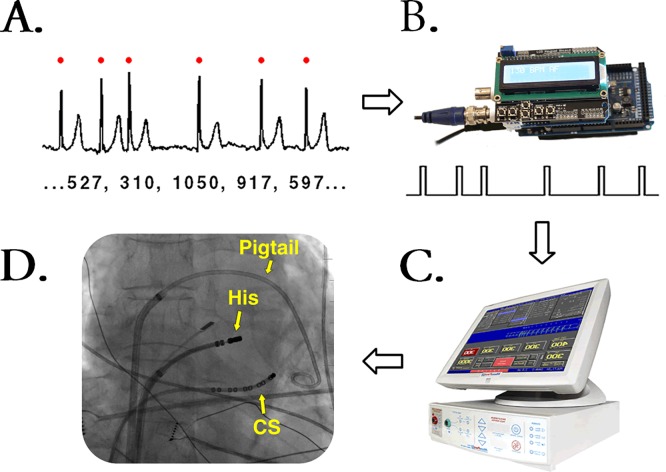
General principle of the method for simulation of AF. (A) QRS complexes were identified in an Holter ECG recording of AF to obtain a text string of the corresponding RR intervals. (B) The sequence of RR intervals was loaded to an Arduino-based microcomputer. The microcomputer generated square-wave pulses according to the RR interval sequence. (C) The pulses from the microcomputer were sensed by a cardiac stimulator, which was set to a sense-pace mode. (D) Pacing was performed by catheters in the coronary sinsus (i.e., left atrial pacing) and at the His bundle region. A pigtail cathter was inserted antegradely to measure the LV pressure.

Fig 1 outlines the principle of the AF simulation. A microcomputer with an analog output (Arduino Due, Arduino, Italy) was connected to an analog ECG input (sensing channel) of an external cardiac stimulator (MicroPace III, Micropace EP, USA). The microcomputer was programmed to generate square-wave pulses (2 V / 20 ms) in a cadence dictated by a preloaded sequence of irregular RR intervals (preparation of the RR-intervals is described in the Pacing protocol). The cardiac stimulator was programmed to pace with an output of ~10 V instantaneously after each of the sensed pulses, thus reproducing the template RR-intervals. Patients were paced by standard electrophysiology catheters simultaneously from the coronary sinus (thereby activating the left atrium) and from the area of the His bundle in the right ventricle.

### Study population

Feasibility of the AF simulation was tested in 10 patients without structural heart disease who were undergoing catheter ablation for paroxysmal AF (Table 1). The sample power was estimated based on the previous studies [3, 7]. The pacing protocol was conducted at the end of the ablation procedure after verifying a stable sinus rhythm. The patients remained under conscious sedation with infusion of midazolam and boluses of fentanyl. Chronic medication was withheld on the day of the procedure. The study was approved by institutional ethics committee (The Ethics Committee of the Institute of Clinical and Experimental Medicine, Prague, Czech Republic, #845/14) and all the patients gave written consent with the investigation.

**Table 1:** Characteristics of the study population.

	n = 10
**Age, years**	59 ± 4
**Male gender, n**	8
**CHA2DS2-VASc**	1.5 ± 1
**LV ejection fraction, %**	57 ± 3
**Left atrial volme index, ml/m2**	40 ± 9
**Betablockers, n**	6
**Antiarrhythmic drugs, n**	4
**Heart rate at rest**	61 ± 12

LV = left ventricular

### Cardiac catheterization

Our method of AF ablation and the catheter setup were described in detail elsewhere [9]. Using transfemoral approach, a decapolar catheter for the left atrial pacing was inserted in to the coronary sinus, a steerable mapping catheter for the His pacing was introduced to the right ventricle, and a 6-F fluid-filled pigtail catheter for measurement of the LV pressure was advanced antegradelly to the LV cavity.

The region of the His bundle was first systematically probed by pace-mapping, to ensure stable capture of the conduction system. The catheter position was adjusted to produce QRS complexes that morphologically best resembled the patient’s native QRS complexes. The selected pacing site was tagged on the 3D electroanatomic map (CARTO, Biosense Webster, Israel) to ensure finding of the identical site in case of incidental catheter dislodgement. The catheter-tissue contact was monitored continuously by inspection of local electrograms and QRS morphology.

### Pacing protocol

**Fig 2:**
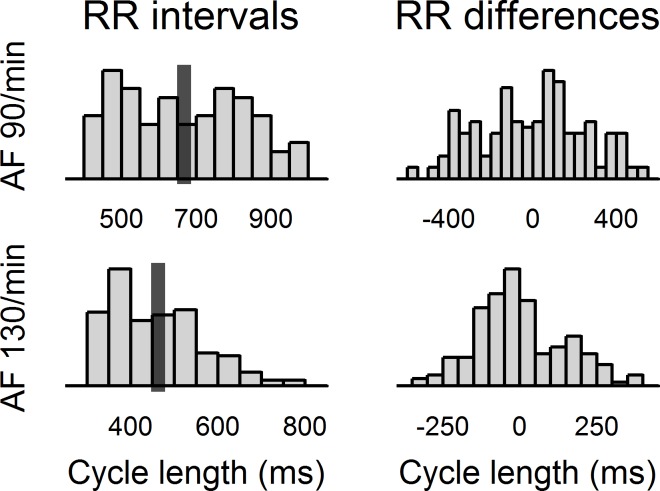
Characteristics of the template AF sequences. The figure shows histograms of the RR intervals and of the absolute differences of the subsequent RR intervals that were contained in the template AF sequences. It can be seen that both sequences contained a broad range of homogenously distributed RR intervals, which enabled to study the effects of heart rhythm irregularity. The dark bars represent mean cycle length (667 ms and 462 ms—i.e., 90/min and 130/min, respectively).

The patients were paced at a mean heart rate of 90/min and 130/min in three 2-minute sequences: regular atrial pacing, regular simultaneous AV pacing, and simulated AF which was represented by the irregular simultaneous AV pacing. The irregular RR-interval sequences were obtained as text files from an open database of processed ECG recordings of patients with AF (The Long-Term AF Database at PhysioNet.org [10]). The text files were scanned by a purpose-made program to look-up representative episodes of AF with the desired mean heart rate and duration (Fig 2). The order of the sequences was randomized for every patient. Each sequence was preceded by an uninterrupted 30-second stabilization period of regular pacing at the same rate–this period was excluded from the hemodynamic analyzes. To assess the intra-individual reproducibility, the whole pacing protocol was repeated after a resting period of 2–3 minutes.

### Data analysis

The LV pressure signal and standard ECG leads were recorded at 1 kHz using a data acquisition device (PowerLab 16/35, ADInstruments, UK). The recordings were analyzed offline in a dedicated software (LabChart 7, ADInstruments). Statistical analyses were conducted in R (www.R-project.org). Data are reported as mean ± standard deviation. P-values <0.05 were regarded significant.

LV systolic pressure, enddiastolic pressure, maximum dP/dt, and Tau were determined beat-by-beat, and the values were averaged over each entire sequence. Estimated error of our catheter system for the measurement of the first derivate of the LV pressure was <10% [11, 12]. The averaged hemodynamic indices were compared between regular atrial pacing and regular simultaneous AV pacing (to evaluate the impact of lost atrial kick), between regular simultaneous AV pacing and simulated AF (to evaluate the impact of heart rhythm irregularity), and between the slower and the faster atrial pacing (to evaluate the impact of a fast heart rate) using a paired t-test with Holm’s correction for repeated measurements [13].

Intra-individual reproducibility of the averaged hemodynamic indices during repeated AF simulation was evaluated by a paired t-test. Beat-by-beat reproducibility of the LV systolic pressure was evaluated by the Pearson’s correlation of pooled data from all patients at both heart rates. At last, QRS complexes were identified by combination of amplitude and derivative criteria, and the average width of the paced QRS complexes was compared by a paired t-test with the width of native QRS.

## Results

**Fig 3:**
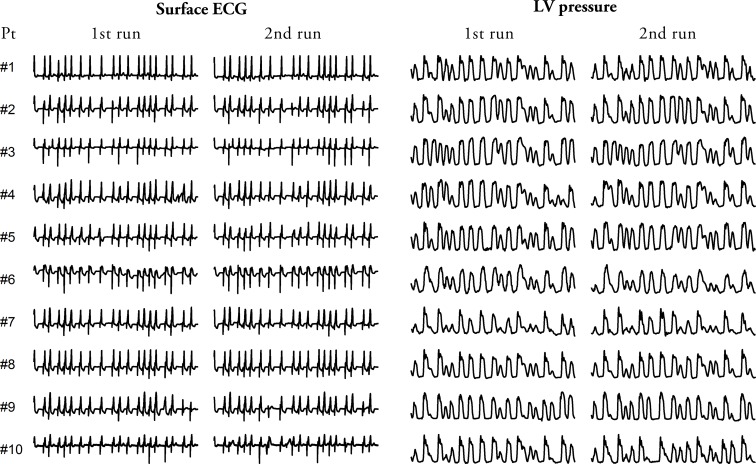
Sample of ECG with corresponding LV pressure signal during simulated AF. The figure shows a 10s sample of ECG and a corresponding LV pressure tracing recorded in all the study patients during two separate runs of the simulated AF. It can be appreciated that (i) the His pacing generated narrow QRS complexes thus indicating intrinsic activation of the ventricles, (ii) the RR intervals followed the same pattern in all the patients at both runs, (iii) the simulated AF generated similar LV pressure tracings among all the patients, (iv) the LV pressure tracings were almost identical within each patient at the two separate runs. Pt = patient.

The study protocol was completed in all patients without procedural complications. Fig 3 shows a 10-second sample of the ECG with matched LV pressure signal recorded during the simulated AF in each of the patients. It can be seen that the irregular AF pacing generated beat-by-beat variations in the LV systolic pressure which resembled the variability of the systolic pressure that occurs in a real AF. Pacing by the same sequence produced similar LV pressure waveforms among all the patients and almost identical LV pressure waveforms within each individual patient. Notably, the paced QRS complexes remained relatively narrow in all the patients (paced vs. native QRS, 126 ± 11 vs. 97 ± 8 ms, p<0.001).

**Fig 4:**
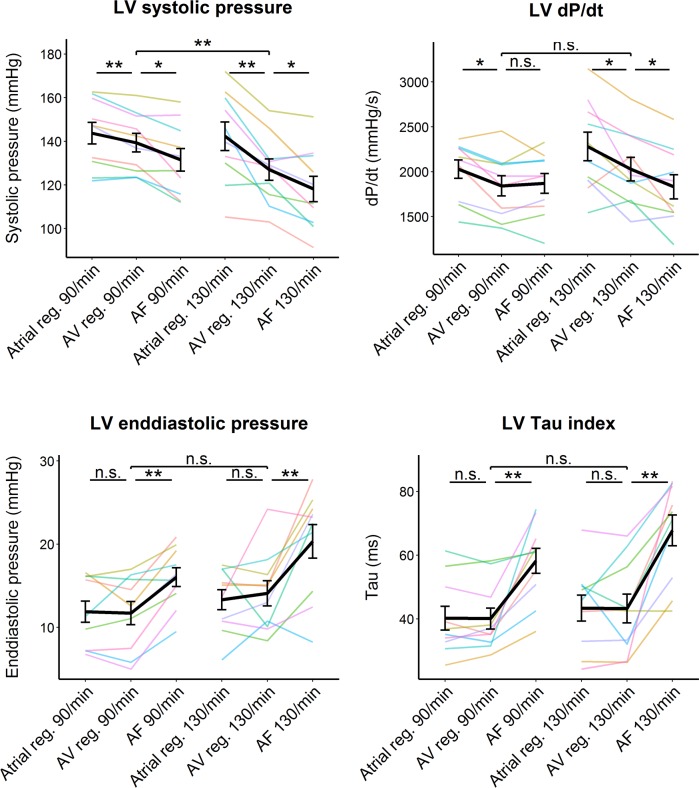
Effect of the simulated AF on the LV performance. The colored lines represent average values in the individual patients during three different pacing modes (atrial regular [reg], simultaneous atrioventricular [AV], simulated AF [AF]) at two different heart rates (90/min and 130/min). The black line with the errorbars represents mean and standard errors. */**/*** = P-value <0.5/<0.01/<0.001 by paired t-test with Holm’s correction; n.s. = non-significant difference (P-value > 0.5);

**Fig 5:**
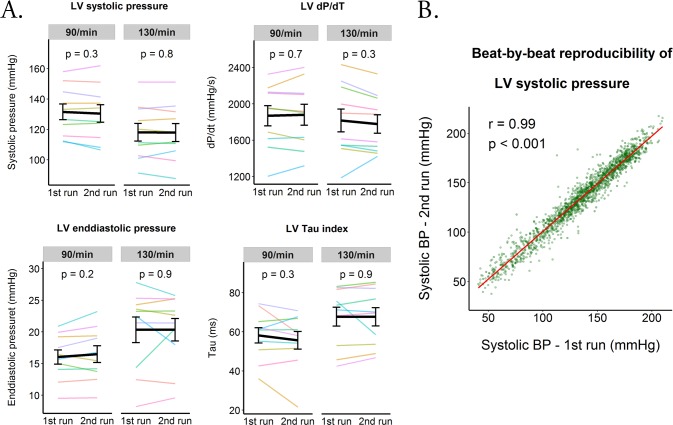
Reproducibility of the LV hemodynamics during two separate runs of simulated AF. (A) The colored lines represent average values in the individual patients obtained at two separate runs of the simulated AF. The black line with the errorbars represent mean and standard errors of all the patients. The p values were obtained by a paired t-test. (B) The graph shows excellent correlation between LV systolic pressure measured in the same patient during two separate runs of simulated AF. The data points were pooled from all the patients.

Table 2 and Fig 4 show the impact of the various hemodynamic components of the simulated AF on the LV performance. The loss of the left atrial contribution to the LV filling caused significant decrease in the LV systolic pressure and dP/dt_Max_ (at both heart rates) but it did not significantly affect the LV enddiastolic pressure or Tau. The irregularity of the heart rhythm itself significantly impaired both the systolic and diastolic LV function, though the dP/dt _Max_ decreased only at the higher heart rates. The fast heart rate caused significant decrease in the systolic pressure but it did not affect any other LV hemodynamic characteristics. Compared to atrial pacing, the simulated AF significantly impaired all the LV hemodynamic characteristic at both heart rates. At last, Fig 5 shows that two separate runs of AF simulation by the same sequence could reproduce similar LV hemodynamics.

**Table 2:** Effect of the simulated AF on the LV performance.

N = 10	Pacing mode			Relative differences		
	Regular atrial	Regular AV	AF	reg. AV vs. reg. atrial	AF vs. reg. AV	AF vs. reg. atrial
**HR 90/min**						
**Systolic BP (mmHg)**	144 ± 16	139 ± 14	132 ± 16	-3% **	-6% *	-9% **
**dP/dT**_**Max**_ **(mmHg/s)**	2027 ± 324	1842 ± 354	1869 ± 352	-9% *	2%	-8% *
**EDP (mmHg)**	12 ± 4	12 ± 4	16 ± 4	-2%	37% **	35% **
**Tau (ms)**	40 ± 12	40 ± 11	58 ± 12	0%	45% **	45% **
**HR 130/min**						
**Systolic BP (mmHg)**	142 ± 21	127 ± 16	118 ± 18	-11% **	-7% *	-17% ***
**dP/dT**_**Max**_ **(mmHg/s)**	2280 ± 503	2029 ± 415	1832 ± 426	-11% *	-10% *	- 20% ***
**EDP (mmHg)**	13 ± 4	14 ± 5	20 ± 6	6%	44% *	52% ***
**Tau (ms)**	43 ± 13	43 ± 14	68 ± 15	0%	56% **	56% **

*/**/*** = P-value <0.5/<0.01/<0.001 by paired t-test with Holm’s correction. AF = simulated atrial fibrillation, AV pacing = simultaneous atrioventricular pacing (from the coronary sinus and the His bundle), BP = blood pressure, reg. = regular pacing.

## Discussion

We have demonstrated hemodynamic feasibility and reproducibility of a novel method for simulation of acute hemodynamics during human AF. The method has three key features: First, custom series of RR intervals—which can be obtained by processing of a real AF recording or created from scratch in a text editor—can be accurately reproduced by pacing in other individuals. This enables a rigorous study of the individual hemodynamic effects of an excessive heart rate and ventricular rhythm irregularity. Second, the His bundle pacing approximates an intrinsic ventricular depolarization, thereby allowing an unbiased study of the cardiac performance [14]. Finally, the simultaneous AV pacing suppresses an unpredictable hemodynamic impact of dissociated sinus rhythm and at the same time obliviates the atrial contribution to the ventricular filling, analogically to AF. This allows to study the impact of the lost atrial contraction in various clinical settings.

### Previously used hemodynamic models of AF

Previous studies investigated the hemodynamics in AF mainly by performing measurements before and after a cardioversion to sinus rhythm [15, 16]. This approach only allowed assessment of the gross differences between both rhythms.

Daud et al. studied the effects of the irregularity of the ventricular rhythm on the cardiac output by comparing regular pacing with pacing by a loop of five extrastimuli with variable cycle length [3]. Of note, commercially available cardiac stimulators usually do not permit to set a longer loop. Such short repetitive sequences, however, were unable to mimic the heart rate variability of a real AF.

Melenovsky et al. used a specially designed external stimulator which delivered random pulses through exposed electrodes of a biventricular pacemaker [7]. The biventricular pacing prevented the acute reduction of the left ventricular function that occurs during right ventricular pacing [17], thus allowing an unbiased study of the cardiac performance. However, this approach was obviously applicable only to the patients indicated for resynchronization therapy.

Our method was in part inspired by the studies of Clark et al. and Segerson et al. [2, 4]. The authors triggered a cardiac stimulator by a playback of an audio recording of a sequence of irregular RR intervals, which was either computer-generated or recorded from a patient with AF [2]. Finally, Obel et al. used a programmable external transcutaneous stimulator that triggered an indwelling pacemaker set to a sense-pace (VVT) mode [8]. Unfortunately, the details about the device have not been revealed.

Our method does not require any special hardware but a low-cost (~40 USD) microcomputer based on the wide-spread Arduino platform. Setting-up of the device does not require a deep knowledge of engineering. The series of RR intervals can be adjusted according to the needs in a common text editor and subsequently simply copy-pasted as a string array to the control software. This feature provides a particularly productive framework when combined with a database of annotated ECG recordings such as the PhysioNet [10]. Our method is not limited to the patients with a sustained AF, complete AV block or implanted pacemaker but it can be applied in all the patients that do not have general contraindications to cardiac catheterization. Finally and most importantly, our model generated meaningful changes in the LV hemodynamics, which were in agreement with the observations found in the previous studies [2, 3, 7].

### Study implications

Our hemodynamic model of AF provides broad opportunities for studying various hemodynamic consequences of AF. For example, it can be exploited to identify which of the attributes of an AF—the fast heart rate, the irregularity of the rhythm or the loss of the atrial contraction—has the dominant role in a particular clinical setting. Such studies could provide data for future tailoring of patient-specific therapeutic strategies: rhythm control by drugs or catheter ablation versus rate control by drugs or AV junction ablation. Also, the hemodynamic model can be used to investigate whether the beat-to-beat variations in the blood pressure that occur in AF can disturb baroreflex or perfusion of organs, in which the blood flow is closely auto-regulated in response to the instantaneous perfusion pressure–particularly in the brain, kidney and myocardium.

### Limitations

An inherent limitation of our method is that the paced sequence must be always faster than the patients’s intrinsic heart rate. Although all study participants fulfilled this condition, some other individuals might need bradycardizing medication during the experiment. Our model of AF would have been even more realistic by pacing independently from the His bundle and at the same time rapid random pacing from the coronary sinus in order to completely abolish the atrial contraction. However, such setup would require a complete AV block or significant bradycardia induced by administration of AV-nodal blocking agents. Moreover, rapid pacing from the coronary sinus could induce AF and thereby compromise the experiment.

The hemodynamic feasibility of our AF model could be even better demonstrated if we reproduced LV hemodynamics assessed during the patient’s own AF. This would require additional measurements and on-line processing of the ECG recordings. Regrettably, such study protocol was not permitted by our institution’s Ethical Committee as it would significantly prolong the whole ablation procedure. On the other hand, the advantage of our protocol was that it allowed to evaluate the inter-individual reproducibility of the LV hemodynamics and, more importantly, it allowed to control the heart rate and heart rhythm irregularity. At last, our model of AF enabled to study only acute changes in the hemodynamics.

## References

[1] Naito M, David D, Michelson EL, Schaffenburg M, Dreifus LS. The hemodynamic consequences of cardiac arrhythmias: evaluation of the relative roles of abnormal atrioventricular sequencing, irregularity of ventricular rhythm and atrial fibrillation in a canine model. Am Heart J. 1983;106(2):284–91. 6869209 10.1016/0002-8703(83)90194-1

[2] Clark DM, Plumb VJ, Epstein AE, Kay GN. Hemodynamic effects of an irregular sequence of ventricular cycle lengths during atrial fibrillation. J Am Coll Cardiol. 1997;30(4):1039–45. 9316536 10.1016/s0735-1097(97)00254-4

[3] Daoud EG, Weiss R, Bahu M, Knight BP, Bogun F, Goyal R, et al Effect of an irregular ventricular rhythm on cardiac output. Am J Cardiol. 1996;78(12):1433–6. 8970422 10.1016/s0002-9149(97)89297-1

[4] Segerson NM, Sharma N, Smith ML, Wasmund SL, Kowal RC, Abedin M, et al The effects of rate and irregularity on sympathetic nerve activity in human subjects. Heart Rhythm. 2007;4(1):20–6. 10.1016/j.hrthm.2006.09.017 17198984

[5] Lavy S, Stern S, Melamed E, Cooper G, Keren A, Levy P. Effect of chronic atrial fibrillation on regional cerebral blood flow. Stroke. 1980;11(1):35–8. Epub 1980/01/01. . 7355427 10.1161/01.str.11.1.35

[6] Friedman HS, O'Connor J, Kottmeier S, Shaughnessy E, McGuinn R. The effects of atrial fibrillation on regional blood flow in the awake dog. Can J Cardiol. 1987;3(5):240–5. 3607591

[7] Melenovsky V, Hay I, Fetics BJ, Borlaug BA, Kramer A, Pastore JM, et al Functional impact of rate irregularity in patients with heart failure and atrial fibrillation receiving cardiac resynchronization therapy. Eur Heart J. 2005;26(7):705–11. 10.1093/eurheartj/ehi066 15618039

[8] Obel OA, Luddington L, Maarouf N, Aytemir K, Ekwall C, Malik M, et al Effects of ventricular rate and regularity on the velocity and magnitude of left atrial appendage flow in atrial fibrillation. Heart. 2005;91(6):764–8. 10.1136/hrt.2003.030940 15894771 PMC1768920

[9] Sramko M, Peichl P, Wichterle D, Tintera J, Maxian R, Weichet J, et al A novel biomarker-based approach for the detection of asymptomatic brain injury during catheter ablation of atrial fibrillation. J Cardiovasc Electrophysiol. 2014;25(4):349–54. 10.1111/jce.12325 24238018

[10] Goldberger AL, Amaral LA, Glass L, Hausdorff JM, Ivanov PC, Mark RG, et al PhysioBank, PhysioToolkit, and PhysioNet: components of a new research resource for complex physiologic signals. Circulation. 2000;101(23):E215–20. 10851218 10.1161/01.cir.101.23.e215

[11] Glantz SA, Tyberg JV. Determination of frequency response from step response: application to fluid-filled catheters. Am J Physiol. 1979;236(2):H376–8. 420321 10.1152/ajpheart.1979.236.2.H376

[12] Falsetti HL, Mates RE, Carroll RJ, Gupta RL, Bell AC. Analysis and correction of pressure wave distortion in fluid-filled catheter systems. Circulation. 1974;49(1):165–72. 4808837 10.1161/01.cir.49.1.165

[13] Holm S. A Simple Sequentially Rejective Multiple Test Procedure. Scandinavian Journal of Statistics. 1979;6(2):65–70.

[14] Mabo P, Scherlag BJ, Munsif A, Otomo K, Lazzara R. A technique for stable His-bundle recording and pacing: electrophysiological and hemodynamic correlates. Pacing Clin Electrophysiol. 1995;18(10):1894–901. 8539158 10.1111/j.1540-8159.1995.tb03838.x

[15] Orlando JR, van Herick R, Aronow WS, Olson HG. Hemodynamics and echocardiograms before and after cardioversion of atrial fibrillation to normal sinus rhythm. Chest. 1979;76(5):521–6. 498823 10.1378/chest.76.5.521

[16] Shapiro W, Klein G. Alterations in cardiac function immediately following electrical conversion of atrial fibrillation to normal sinus rhythm. Circulation. 1968;38(6):1074–84. 5725769 10.1161/01.cir.38.6.1074

[17] Lieberman R, Padeletti L, Schreuder J, Jackson K, Michelucci A, Colella A, et al Ventricular pacing lead location alters systemic hemodynamics and left ventricular function in patients with and without reduced ejection fraction. J Am Coll Cardiol. 2006;48(8):1634–41. 10.1016/j.jacc.2006.04.099 17045900

